# The energy model of insulin resistance: A unifying theory linking seed oils to metabolic disease and cancer

**DOI:** 10.3389/fnut.2025.1532961

**Published:** 2025-04-29

**Authors:** Catherine Shanahan

**Affiliations:** Rebel Well, LLC, Orlando, FL, United States

**Keywords:** mitochondria, seed oil, PUFA, oxidative stress, cancer, insulin resistance, Warburg effect, obesity

## Abstract

The problem of insulin resistance has exploded in recent decades, from practically nonexistent in 1950, to nearly ubiquitous today. Despite this, the dietary origins of insulin resistance remain elusive. Many have identified the Western Diet, focusing on saturated fat. However, population-scale consumption data shows that our consumption of saturated fat has remained unchanged, while our consumption of polyunsaturated fats has increased by more than 300%. This paper discusses the primary source of those polyunsaturated fatty acids (PUFA), a collection of eight chemically similar refined, bleached, and deodorized (RBD) seed oils, i.e., soy and canola, that now, together, represent the number one source of calories in the United States today, or approximately 30 percent of the average person’s daily intake. The Energy Model of Insulin Resistance hypothesizes that RBD seed oil consumption can promote cellular oxidative stress, forcing cells to change their fueling strategy to reduce oxidative stress. This is accomplished by increasing aerobic glycolysis to minimize fat oxidation. Observed in both cancerous and insulin resistance cells, aerobic glycolysis is also known as the Warburg Effect. While beneficial to individual cells, at the whole-organism level, it disrupts intravascular glucose homeostasis, ultimately elevating insulin and counter-regulatory hormones (CRH) simultaneously. CRH oppose the insulin signal, leading to the phenotype of insulin resistance. In summary, the Energy Model of Insulin Resistance provides a framework for understanding that the primary metabolic deficit in people with insulin resistance may not be abnormal insulin signaling, but rather an abnormally increased metabolic demand for sugar. If correct, this would elucidate the mitochondrial origins of the Warburg Effect and suggest that avoiding RBD oils represents an important and understudied dietary strategy for addressing insulin resistance and cancer.

## Introduction: the unexplained rise of insulin resistance

Metabolic health is at an all-time low, hovering just under 7% of US Adults (1). According to the latest NHANES dataset, fewer than 25% of adolescents have an ideal HOMA-IR score of 1 or less (2). IR often progresses to type 2 diabetes. But it is also associated with many disabling and fatal conditions even before this progression has occurred. In spite of its prevalence and devastating consequences, the dietary root cause and cellular mechanism of insulin resistance remain unclear.

Various models have been proposed, each with it is own shortcomings from failing to be fully supported by epidemiological or mechanistic considerations. I will highlight the most important models here.

Perhaps the most enduring model of insulin resistance proposes that obesity itself drives insulin resistance. But this fails to explain the increasing prevalence of insulin resistance in normal weight individuals (3). Lack of exercise has also been put forth as a driver of insulin resistance, but this does not explain how athletes become insulin resistant (4). It is notable that both obesity and lack of exercise are thought to cause insulin resistance by some combination of inflammation and oxidative stress (5).

The Western Diet has also been identified as a causative agent, again linked to oxidative stress (6, 7). In terms of macronutrients (as opposed to minor constituents such as pesticide residue and artificial coloring agents), the focus is generally on fat and sugar. However more attention is generally paid to fat, especially saturated fat, than sugar, and this is reflected in the dietary guidelines for Americans, which place caps on total fat and saturated fat intakes that must be adhered to in order for any institution to qualify for federal funding. No similar sugar intake cap exists (8). Additionally, articles on the Western diet such as those cited in the above two review articles more frequently discuss animal experiments where insulin resistance was induced by high fat feeding than by high sugar feeding.

In contrast to those focusing on fat as the driver of the oxidative stress inducing insulin resistance, proponents of the newer carbohydrate model of insulin resistance focus on carbohydrate-driven oxidative stress. They hypothesize that refined sugar and carbohydrates may elevate insulin too often, such that they “wear out” the body’s ability to respond to insulin (9). In this case, oxidative stress is provoked by elevated serum glucose (10). However, by definition blood glucose levels do not exceed 200 mg/dL for any significant period of time until insulin resistance has *already* progressed to type 2 diabetes, calling this mechanism to question.

### RBD seed oils: an overlooked component of the western diet

The controversy over carbohydrate versus fat as a causative agent may exist in part because the characterization of the Western Diet is fundamentally flawed. Clemente-Suárez et al., write in 2023 that the Western Diet is “…a modern dietary pattern that is characterized by high intakes of processed and refined foods, red and processed meats, added sugars, and *saturated and trans* fats [emphasis mine]….” (6) Other authors make similar inaccurate remarks about saturated fat and trans fat (7, 11). While trans-fats had increased in the US diet during the 20th century, since 2000 they have declined as a result of the trans-fat ban, and by 2018 were largely eliminated (12). Secondly, and more pertinent, our saturated fat consumption is not increased compared to what it was before the incidence of metabolic disease began its precipitous rise in the middle of the 20th century. According to Figure 20, of the USDA publication Nutrient Content of the US Food Supply, 1909–2000 (13), our saturated fat consumption has remained remarkably stable in that time period, hovering between 50 and 58gm per day per person. However the same figure shows that our consumption of polyunsaturated fats increased nearly 300% between 1909 and 2000. The primary source of these polyunsaturated fats is the collection of eight refined, bleached, deodorized (RBD) seed oils that are the focus of this article and that remain critically under examined as a source of the oxidative stress (and inflammation) that many authors agree drives insulin resistance.

### Oxidative stress: the crucial common theme?

From a mechanistic standpoint, many existing models of insulin resistance share the common theme of oxidative stress, as I briefly outlined above. These authors typically invoke inflammation as well as oxidation, as if the two processes are unrelated and must be examined independently. Others have made the same kinds of statements, and it seems to be a prevailing assumption that the two processes are independent (14–16). However this is not quite accurate.

It is important to recognize that inflammation generally begins with oxidative insults to the cell, particularly cell membrane phospholipids, whose oxidation initiates the activation of eicosanoids that drive the inflammatory cascade (17–19). If inflammation is only a secondary response to a primary insult, as appears may be the case in many if not all scenarios, then it may be unnecessary to invoke inflammation as a separate root cause driver of insulin resistance, and more logical to focus on oxidative process.

Indeed, Denham Harmon identified free radicals and their resultant oxidative insults as the root cause of cellular damage and aging in 1954 (20). The theory has been modified over the years, but the general thrust is the same: oxidative damage drives disease, inflammation, and aging (21). Thus, it is highly relevant that RBD seed oils are uniquely susceptible to free radical damage.

## RBD seed oil, a novel food for humans

### What is RBD seed oil?

The majority of edible oils in our food supply undergo refining, bleaching, and deodorizing after extraction. The edible oil industry refers to these as RBD oils. The term seed oil does not have an industry definition. For the purposes of this article, the term “RBD seed oil” will refer to the collection of eight vegetable seed oils with the highest concentrations of easily oxidized polyunsaturated fatty acids: corn, canola, cottonseed, soy, sunflower, safflower, rice bran, and grapeseed. Palm, coconut, peanut, olive and avocado can also be RBD but are lower in PUFAs and thus less of a concern.

These eight oils collectively comprise up to 80 percent of the average American’s fat calories and nearly one-third of their total caloric intake (22). Any food constituting such a large portion of the daily caloric intake demands scrutiny, particularly a novel food that has no precedent of safe use by human prior to it is widespread introduction into the food supply during the 20th century.

**Figure 1:**
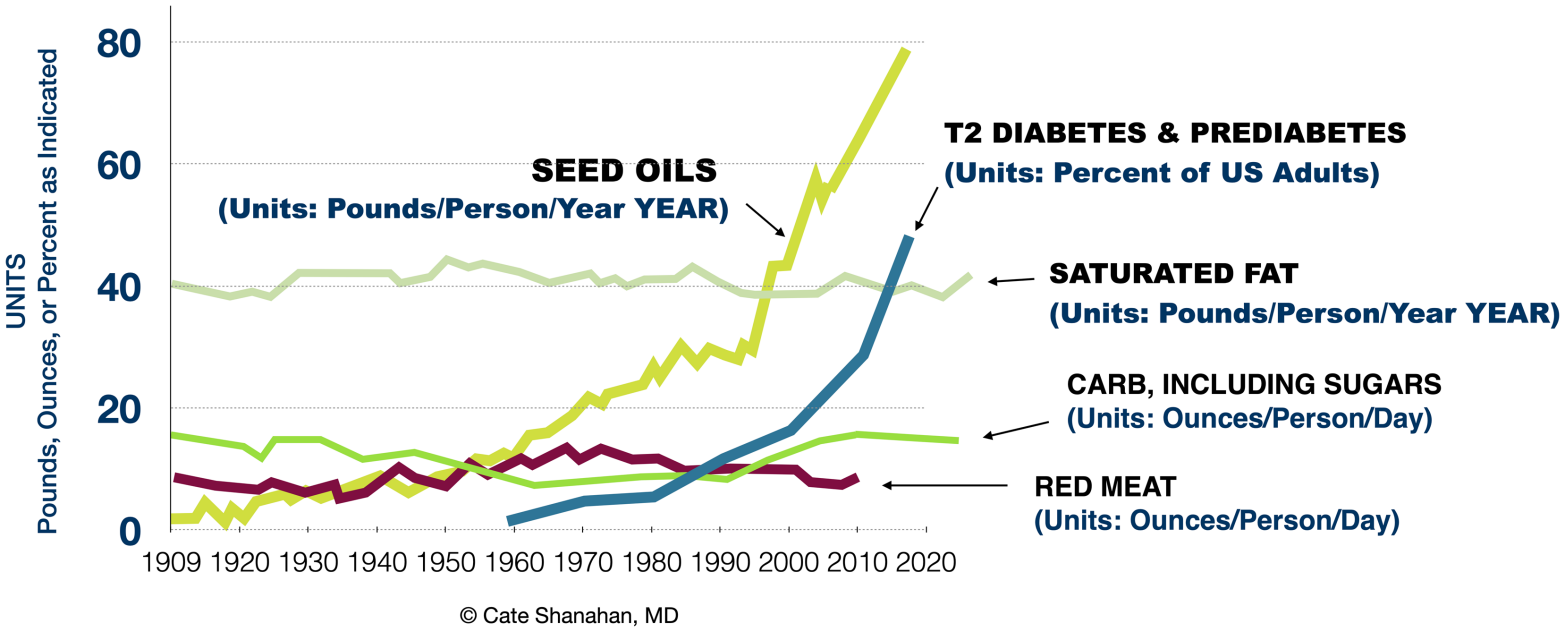
Associations between major dietary components and rates of advanced insulin resistance in the US population from 1909 to 2020.

Notably, over the last century we can appreciate a striking similarity between the rise of RBD oil consumption and the rise of metabolic disease, as illustrated in Figure 1. The similarity is greater than that of sugar, which has more often been implicated as a root cause of metabolic disease.

### Recommendations to consume RBD oils

During the 20th century, the prevailing public health advice was to reduce saturated fatty acid and increase polyunsaturated fatty acids (PUFA). This emerged from the assumption that, because saturated fat consumption elevates cholesterol, that necessarily means it also causes heart attacks. Notably, the American Heart Association recommended these oils starting in 1961, but preliminary data from the first human trial to test this idea was not even collected until 1963, and not published until 1966 (23, 24). This is remarkable, and worthy of more attention that it has received, given the AHA’s claims that it is an evidence based organization.

Since the AHA first made its recommendation, multiple reviews have found no evidence of increased cardiac mortality from saturated fat (25–29) and the same holds for elevated cholesterol (30–32). Although this evidence significantly undermines the saturated-fat-cholesterol theory, the Dietary Guidelines (DGA) are based on low quality evidence and expert opinion (33, 34). Although calls have been made for the DGA to use higher quality evidence, the experts involved assert that there is enough evidence that saturated fat does increase cardiac mortality (35). As a result, the Dietary Guidelines for Americans continue to make the same recommendation to consume more polyunsaturated oils, as they have since first published in 1980. And without a cap on their consumption, our dietary intakes of RBD seed oils have trended upwards for decades (see Figure 1). Today, the average American gets the majority—approximately 80 percent—of their fat calories from these eight RBD seed oils.

It is also remarkable that these oils were initially manufactured for purposes other than human consumption and never tested on any scale before being released into the food supply (36). Given their widespread consumption and the lack of long term safety data, it is critically important to understand that their chemical makeup is unique among food ingredients in several key aspects that must be considered together to understand their impact on human metabolic health.

### RBD seed oil’s novel chemistry

**Figure 2:**
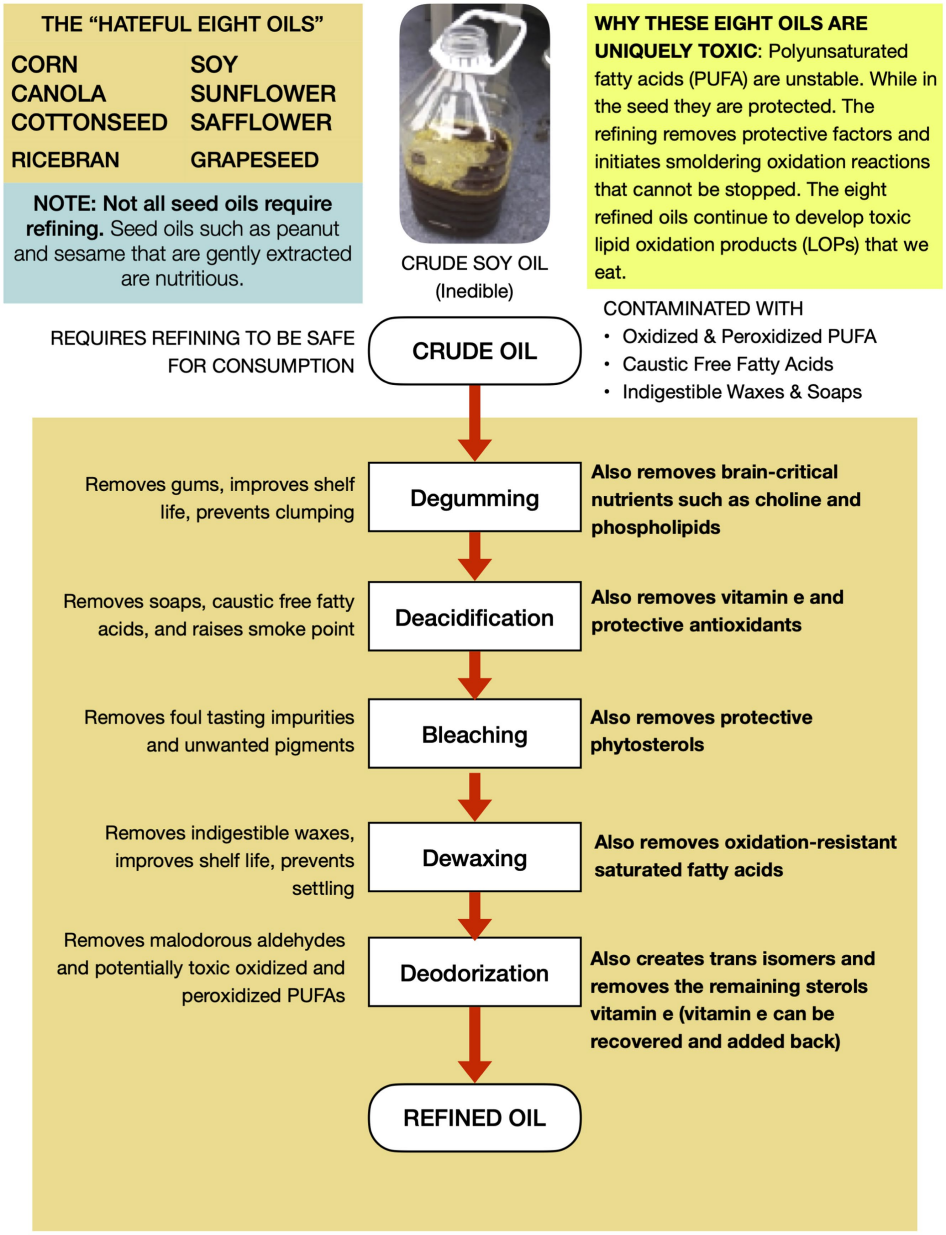
RBD seed oils are uniquely non-nutritive and easily oxidize into aldehydes and other toxins.

RBD seed oils are chemically distinct from fats and oils humanity has typically consumed in several aspects that pertain to their potential health effects. Unfortunately, dietitians and other nutrition experts often lump all “plant based” oils together, sending the strong message that if a consumer cannot afford more expensive extra virgin olive oil, they can simply purchase an RBD seed oil and get equivalent nutrition and expect the same health outcomes. This is erroneous. Four differentiators that pertain to their potential to promote significant redox imbalance will be highlighted here, and summarized in Figure 2.

#### Higher PUFA content

When compared to traditional fats, these new oils have less saturated fat and more easily oxidizable polyunsaturated fat, primarily linoleic acid. This reflects the fatty acid profile of the seeds from which they are derived. The eight seed oils mentioned above contain from 25 to 75 percent PUFA fatty acids. The predominant PUFA is the 18 carbon omega-6 linoleic acid, although canola also contains approximately 10 percent omega-3 linolenic. Soy oil is the dominant RBD seed oil in the US food supply representing 80 to 90 percent of all RBD seed oils consumed (37). It contains approximately 55 percent polyunsaturated fatty acids. Butter, tallow, lard, chicken fat, coconut, and olive were previously the dominant fats in the American food supply. Their PUFA content ranges from a low of 4 percent for tallow, and a high of 20 percent for chicken fat.

#### Inedible when first extracted

In contrast to the first press of other culinary oils like olive, sesame, and coconut, the crude oil that is extracted during the industrial processing of the eight aforementioned oilseeds is initially unsuitable for human consumption due to “process contaminants” such as free fatty acids, waxes, soaps, and oxidized lipids. Thus crude seed oils must be refined to remove those unwanted elements (38). Unfortunately, refining also removes important nutrients that impact human health significantly.

#### Lower antioxidant and vitamin content

The second differentiator is an unintended but unavoidable byproduct of the processing that the vast majority of RBD seed oils consumed undergo, which reduces their nutrient content significantly. Unlike other oils, which reflect the nutritional profile of the seeds from which they are derived, RBD seed oils do not. Nutrients lost to oil refining vary by type but include a 25–35% reduction in soybean oil tocopherols (39, 40), a 60% reduction in Canola oil polyphenols (41), 99.8 percent loss of soybean oil phospholipids (39, 40). 90.7 percent loss of iron (39), and significant losses of copper, iron, magnesium, and calcium (40). Some of these nutrients function to protect the oil from oxidation, and their removal during refining makes the PUFA more susceptible to oxidation, with important consequences to their toxicity, discussed next.

#### Lipid oxidation products

PUFA oxidize more readily than saturated and monounsaturated fatty acids, and the resultant products are broadly called “lipid oxidation products.” As a result, bottles leaving the factory contain a variety of partly oxidized PUFA that often have toxic properties (42). The concentration of toxins increases over time due to the removal of stabilizing factors, the small amount of oxygen and the fact that many lipid oxidation products (LOPs) are themselves more susceptible to oxidation than the native linoleic and linolenic molecules (43, 44).

Lipid oxidation products can be quite toxic. Two of the most well studied LOPs are 4HHE and 4HNE. 4HHE is derived from oxidation of the omega-3 alpha-linolenic acid, and 4HNE is derived from oxidation of the omega-6 linoleic acid (45, 46). Alpha-beta unsaturated aldehydes represent another important class of LOPs present in RBD seed oils. Martin Grootveld and his associates measured the concentration of alpha-beta unsaturated aldehydes that form during shallow pan frying for 30 min as well as in fast food deep fryers subject to continuous heating for several days and found levels of both exposures to be similarly high. They detected crotonaldehyde at levels of 1.8–5.7 mg, and n-hexanal at levels of 2.5–9.5 mg. To help readers conceptualize the potential health hazard, he explained that these levels are “not dissimilar” to the alpha-beta unsaturated aldehyde exposure from “smoking of a (daily) allocation of 25 tobacco cigarettes” (47, 48).

With these considerations in mind, let us now return to the hypothesis.

## The energy model of insulin resistance

**Figure 3:**
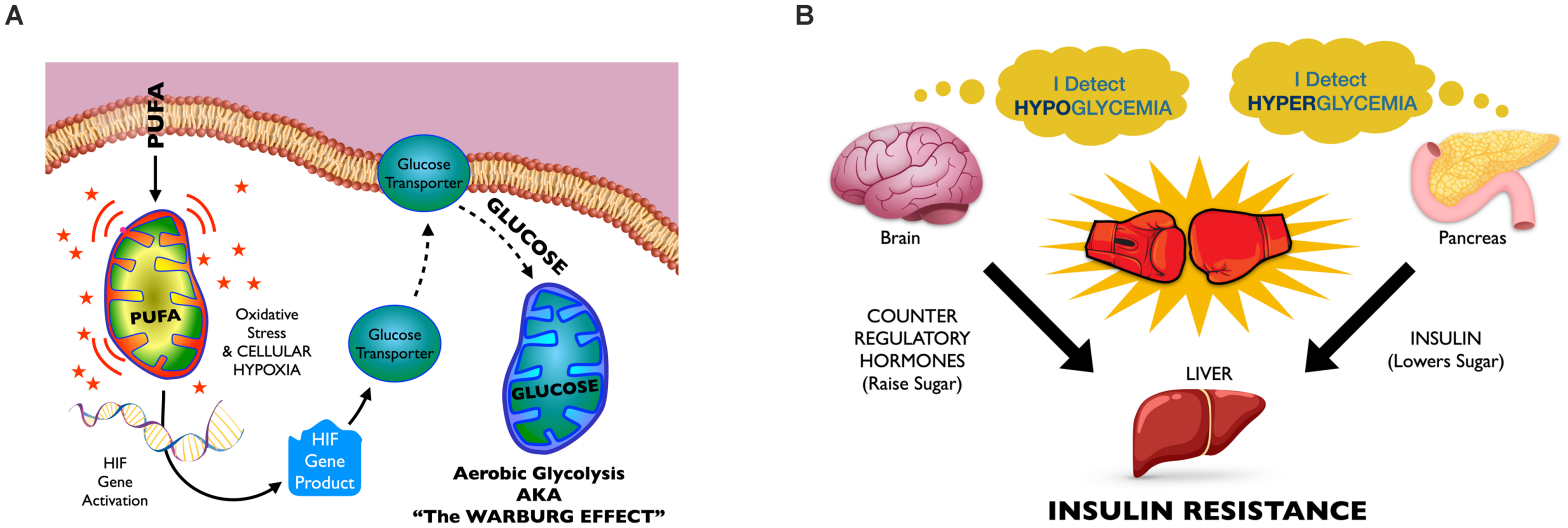
The energy model of insulin resistance: **(A) The intracellular level. Mitochondrial PUFA oxidation harms cell structures, causing a shift to glucose oxidation as a protective measure**. High-PUFA oils build high-PUFA body fat, and their mobilization between meals likely increases intracellular PUFA concentrations above historical norms. Mitochondrial PUFA oxidation promotes more oxidative stress than does oxidation of more saturated fatty acids. Oxidative stress, in turn, can promote periods of hypoxia that, among other effects, activate hypoxia inducible factor (HIF) genes. HIF genes increase GLUT-1 production, increasing mitochondrial glucose oxidation. **(B) The tissue level. Due to the excessive glucose utilization by cells throughout the body, blood glucose levels start to decline, causing subclinical hypoglycemia**. Hypoglycemia triggers counter regulatory “stress” hormone release and glucose elevations that can stimulate pancreatic insulin release. However, the presence of stress hormones blunts insulin’s ability to tell the liver to stop elevating glucose, thus the liver “resists” insulin signal. As insulin resistance progresses, the brain “defends” higher levels of glucose, and hyperglycemia predominates.

The Energy Model of Insulin Resistance is a multi-disciplinary hypothesis that draws connections between the recent changes to our food supply that have produced alterations in human adipose fat, and their likely impacts upon cellular redox homeostasis that could lead to altered whole-body glucose homeostasis and ultimately insulin resistance (see Figure 3 for a visual guide through the process.). The model posits that RBD seed oil consumption contributes to significantly altered adipose composition such that cells metabolizing body fat are likely to experience more oxidative stress than cells metabolizing historically normed adipose fat. Oxidative stress, in turn, induces aerobic glycolysis, also known as The Warburg Effect. Increased glucose utilization at the cell level potentially disrupts whole body glucose homeostasis and culminates in insulin resistance, as will be discussed. Cellular oxidative stress can simultaneously promote inflammatory disease, developmental disease, infertility, and cancer, and represent a unifying mechanism we can invoke to explain the observed links between metabolic disease and the other chronic diseases that are increasing in prevalence in tandem with the obesity epidemic.

Glucose oxidation in the presence of oxygen is called aerobic glycolysis, and is also known as The Warburg Effect. This metabolic state has long been associated with cancer but more recently recognized in insulin resistance. The Energy Model hypothesizes that, when many cells simultaneously transport more glucose into the cell than normal, this can lead to episodes of subclinical hypoglycemia, discussed in Figure 1B.

### The energy model of insulin resistance: a four component hypothesis

This section outlines the four-components of the hypothesis that a shift in our food supply, from traditional fat to novel seed oils, is the primary driver of a shift in our metabolic health from that of normoglycemia to insulin resistance. The four components, described herein, have long been observed and discussed. However, they have not yet been considered together as components of an important story—the story of the rise of metabolic disease during the past 120 years.

The four components are:

- Replacing animal fats with RBD seed oil promotes cellular oxidative stress.
- High seed oil diets produce high PUFA body fat and harm mitochondria.
- Mitochondrial adaptation to increased oxidative stress may involve increased aerobic glycolysis.
- Increased aerobic glycolysis profoundly disrupts glucose homeostasis, leading to insulin resistance.

The four components are inter-related and partly sequential. Components I and II likely represent the initial insults that lead to the development of Components III and IV, the latter representing physiologic adaptations to the initial insults.

Components I and II initiate the cascading sequence of events that lead to insulin resistance by depleting antioxidant capacity, according to the evidence presented below in the sections covering these components. Depleted antioxidant capacity likely has numerous consequences and the one this paper discusses is the possibility that mitochondria become more susceptible to oxidative stress. In the section covering Component III, I will discuss the evidence that oxidative stress leads to increases in aerobic glycolysis, also known as The Warburg Effect. The final component, Component IV, discusses the consequences of supra-normal cellular glucose consumption. Drawing higher than typical levels of glucose from circulation could lead to repeated episodes of subclinical hypoglycemia. This has consequences to multiple tissues that must be considered together in order to comprehend their true impact.

The Energy Model provides a framework for understanding that the important initial defect in insulin resistance is an increased metabolic demand for sugar rather than a decreased sensitivity to insulin. This increased metabolic demand for sugar does not abate as insulin resistance progresses to prediabetes and type 2 diabetes; indeed it appears to accelerate.

Some aspects of the theory are more well supported by direct experimental data than others, and those aspects that have only indirect support will be discussed in additional detail in the next section. Additionally, since saturated fat is more often highlighted as the driver of metabolic disease than is polyunsaturated fat, this topic will also be discussed.

### Component I: Replacing animal fats with RBD seed oils promotes cellular oxidative stress

It is a fundamental principle of chemistry that double bonds are more susceptible to oxidation than single bonds, and that the 1, 4 diene structure of polyunsaturated fatty acids’ double bonds renders them particularly susceptible to oxidative attack compared to monounsaturated and saturated fatty acids (49). To protect those fragile fatty acids from oxygen attacks, biological systems embed membranes with vitamin E. Those membrane structures with higher PUFA concentrations require greater vitamin E concentrations to protect them (50). However, Buettner, G. R., makes it clear in his 1993 publication “The Pecking Order of Free Radicals and Antioxidants” that to protect cell membrane PUFA, antioxidants must work in teams. Vitamin E passes off its radical to vitamin C, which passes off its radical to Glultathione, which must be “recharged” by the enzyme glutathione reductase (51). If any one of the nutrients involved is in short supply, the membrane protection system fails.

Animal experiments support the related idea that as you increase the PUFA content of an animal’s diet without also increasing antioxidants, the tissue levels of antioxidants are likely to decline or become depleted, with increases in oxidative stress. Raederstorff et al. cite experiments showing that as dietary linoleic acid content increases, vitamin E levels in tissues such as liver or plasma drop unless intake is adjusted upward (50). Bazinet and Layne note that in brain tissue, GSH levels often drop acutely under high PUFA conditions due to this oxidative burden (52). These authors also note that chronic PUFA exposure might upregulate GSH synthesis as an adaptation—via pathways like Nrf2 activation—*if* precursors (e.g., cysteine) are not limiting. Chronic PUFA exposure might ramp up GSH production if the system adapts, but if not—like in nutrient deficiency or disease—depletion of just one of the many nutrients involved in any given enzyme system could lead to failure of the entire system. The implication is that a high PUFA diet demands a specific set of nutrients to sustain without which the organisms will suffer harms related to oxidative stress.

A variety of investigators have shown experimentally that high linoleic acid diets induce oxidative stress in humans and experimental animals (53–55). Additionally, human observational studies have linked linoleic acid consumption to inflammatory disorders, obesity, immune disorders, infertility, ALS, psychological disorders (56–58). These authors propose the effects are mediated by oxidative stress.

In 2001, Kanner and Lapidot performed a series of experiments that considered an additional variable of stomach acid. They fed animals soy oil (55–58% PUFA) with meats and found the iron catalyzed Fenton reactions, leading to rapid lipid peroxidation with resultant hydroperoxide and reactive aldehyde production (59). This suggests that not only are fried foods going to increase our exposure to harmful LOPs like hydroperoxides and reactive aldehydes, certain food combinations will do so, too (53, 54, 60).

Having established that seed oils can, at least under certain circumstances, deplete antioxidant capacity in ways that would disrupt redox homeostasis, let us next discuss another consequence of seed oil consumption.

### Component II: High seed oil diets produce high PUFA body fat and harm mitochondria

#### Changes to human adipose PUFA content during the past half century

Numerous animal studies have shown that dietary PUFA concentrates in adipose tissue in proportion to dietary consumption. Scant human studies have been done, but those that have have shown similar results. Guyenet (61) compiled data from studies published between 1955 and 2006 that evaluated the linoleic acid content of human adipose. (Linoleic acid is the predominant PUFA in RBD seed oil.) The data shows that in 1955 year the average linoleic content in adipose tissue ranged from 5 to10%, and by 2008 it rose to over 20%. Hodson et al. found that omega-3 fatty acids, alpha-linolenic, EPA, and DHA represent 0.7–1.0% of total fatty acids, and arachidonic acid accounts for another 0.5–1.0% (62). Current per capita RBD seed oil consumption is likely significantly greater than it was in 2006, due to the replacement of hydrogenated oils by liquid seed oils, thus our adipose total PUFA concentration is likely significantly higher than 20%.

Lipolysis of this adipose between meals releases PUFA into the circulation as free fatty acids to be taken up by cells where it may be utilized as substrates for mitochondrial beta-oxidation, with potentially detrimental effects to mitochondrial function.

#### Evidence that mitochondrial PUFA oxidation induces mitochondrial oxidative stress, apoptosis and ferroptosis

In 2002, researchers from the Institute for Neurosciences in Padua, Italy, demonstrated that just a few minutes of exposure to omega-6 linoleic acid depleted mitochondrial antioxidant capacity, after which time mitochondrial energy production plummeted to less than 50 percent of normal, and apoptosis ensued (63). The effects were more pronounced with omega-3 alpha-linolenic acid. In 2010, Shamato-Nagai et al. similarly demonstrated that PUFAs induce oxidative stress and cell death (64).

In 2012, Dixon SJ et al. identified a form of cell death called ferroptosis. They identified the peroxidation of polyunsaturated fatty acids as a key driver of the oxidative damage to cellular membranes leading to cell death. In 2019, Gao et al. demonstrated that mitochondria play a pivotal role in a subtype of ferroptosis induced by cysteine-deprivation (65). 2018, Picou et al. showed that omega-3 fish oils induced mitochondrial damage and ferroptosis in human derived leukemia cells (66). In 2023, Watts et al. showed that omega-6 and omega-3 fatty acids increase ferroptosis sensitivity in C elegans (67). In 2023, Petan et al. observed that when fat droplets that normally aggregate in cells release polyunsaturated fatty acids too rapidly, that process initiates ferroptosis and, subsequently, results in cell death (68). Also in 2023 Mortensen et al. compiled a review of multiple articles showing that, relative to PUFA, dietary monounsaturated fatty acids reduced susceptibility to ferroptosis (69).

When mitochondrial antioxidant capacity is overwhelmed, oxidative damage accumulates, leading to dysfunction that impacts the entire cell (70, 71). Thus it is incumbent upon the cell to support mitochondrial health, and this may involve changing fueling strategies. This will be discussed next.

### Component III: Aerobic glycolysis and the Warburg effect

In the 1930s, Otto Warburg had observed tumors taking up enormous amounts of glucose compared to the surrounding cells, and suspected it was related to dysfunctional mitochondria. In subsequent decades other investigators noted that cancer cells tended to up regulate glycolysis in aerobic conditions rather than solely in anaerobic conditions. Eventually, the term The Warburg Effect because synonymous with aerobic glycolysis.

In 1977, Sidney Weinhouse, speculated that “The Warburg Effect might protect cells from oxidative damage by limiting mitochondrial respiration under conditions of high metabolic demand.” Although his perspective was that glycolysis was an attempt by the cell to *minimize* oxidative stress, not a result of it. Later investigators, with better instrumentation, were able to flip the script, showing that reactive oxygen species did indeed drive the Warburg Effect (72). Thus, oxidative stress can provoke aerobic glycolysis and if PUFA induce oxidative stress, they can likely induce aerobic glycolysis.

Meanwhile, others were linking PUFA to cancer. Efraim Racker, PhD, a pioneer in mitochondrial research, made an early link between dysfunctional mitochondria and polyunsaturated fatty acids, specifically having observed that polyunsaturates were mitochondrial uncouplers. In a 1963 editorial, he expressed concern that their long term use would likely “give rise to toxic reactions” and potentially cause cancer (73). He was gravely concerned that American Heart Association’s recommendation to swap out saturated fats for high-PUFA oils might prove unsound. In the same editorial, he cautioned that “More extensive studies on the toxicity of unsaturated fatty acids may be in order before their indiscriminate use in foods or drugs is condoned.”

#### Is glycolysis a survival strategy for cells chronically exposed to oxidative stress?

While a thorough investigation of this possibility is beyond the scope of this article, I want to raise the point because, as discussed, RBD oils are changing the chemistry of human body fat and potentially inducing a chronic state of increased oxidative stress. Reverting to glycolysis may be an atavistic survival strategy that many cells adopt in order to limit their exposure to damaging oxidative stress (69, 74). It benefits the individual cell, but ultimately harms the body.

Several lines of evidence have since converged around the notion that PUFAs induce not only oxidative stress but also glycolysis. Cellular oxidative stress induces a response that shifts fuel substrate from fatty acid to glucose. This is mediated by hypoxia-inducible factor (HIF)-1α nuclear accumulation, which activates glycolysis, while suppressing mitochondrial oxidative phosphorylation and fat oxidation (75–80). We have fairly ample evidence that oxidative stress can induce hypoxia inducible factor (HIF) gene expression (81–83). What is more, while the Warburg Effect has long been linked to cancer, newer evidence also links the Warburg effect to insulin resistance (84–86). And finally, patients with type 2 diabetes have increased activity of glycolysis and lactate production (87, 88). All of this taken together should serve to bolster the notion that seed oil consumption can shift cellular fueling strategy in ways that lead to increased blood glucose consumption.

The downstream effects of this will be discussed next.

### Component IV: Increased cellular glucose consumption disrupts glucose homeostasis, leading to insulin resistance

It is self-evident that increased glucose consumption occurring between meals and overnight could potentially promote at least a mild form of hypoglycemia. In the postabsorptive (aka fasted) state, the supply of blood sugar is limited to a teaspoon’s worth of glucose at any given time, roughly16 calories. The circulatory system is simply not capable of distributing these scant calories over thousands of miles of capillaries with perfect efficiency. Ordinarily, mobilized free fatty acids provide cells with energy between meals, preventing blood glucose from dropping below the body’s normal set points. But, the shift from fat oxidation to glycolysis will increase body-wide glucose utilization, potentially causing blood sugar to dip.

The key to understanding the full impact of repeated episodes of subclinical hypoglycemia involves examining how the body’s various organs response to it. The brain vigorously defends against hypoglycemia by releasing counter regulatory hormones (CRH), frequently referred to as “stress hormones,” and the net effect is often slightly elevated glucose (89), leading to the subsequent pancreatic release of insulin. Thus, we have insulin and counter regulatory hormones present simultaneously. CRH, by definition, resist insulin’s signaling—thus leading to the phenotype of insulin resistance: high blood sugar and high insulin occurring simultaneously, and continuously.

Counter regulatory hormones (CRH) fiercely defend blood glucose. Mild drops in blood glucose, to 65–70 mg/dL, can trigger CRH release well before symptoms of more severe hypoglycemia (<50 mg/dL) appear (90, 91). Thus, if enough cells shift to aerobic glycolysis, it could lower glucose enough to stimulate release of CRH, although evidence for this is indirect (see below). Elevated levels of CHR, in turn, can induce mild *hyper*glycemia even in the presence of insulin. Simultaneously elevated insulin and CRH actions at the level of the hepatocyte produce the early stage phenotype of insulin resistance.

Indeed, chronic emotional stress is thought to induce insulin resistance by way of cortisol and catecholamines. In 1993, Surwit et al. (92) proposed that “The effects of stress on glucose metabolism are mediated by a variety of ‘counter-regulatory’ hormones that are released in response to stress and that result in elevated blood glucose levels and decreased insulin action.” Similarly in 2022, Sharma et al. (93) hypothesized that “The release of catecholamines and a rise in serum glucocorticoid concentrations caused by psychological stress enhance the requirement for insulin and insulin resistance.”

Interestingly, hypoglycemia can cause hunger and concentration problems. Over the past few decades, the number of people snacking between meals has increased, and one of the most commonly cited reasons for snacking between meals is inability to concentrate. According to an industry report published by Mintel called “Snacking Motivations and Attitudes U.S. 2015,” 39 percent of millennials report snacking to “stay focused” throughout the day (94). Thus, along with the epidemic of insulin resistance, we may also be in the midst of an unrecognized epidemic of subclinical hypoglycemia.

#### Human experiments showing dietary PUFA promotes hypoglycemia

A number of investigations suggest that PUFA increases the body’s reliance on blood glucose for energy between meals. In 1988, Jones et al. (95) placed participants on either a high- or low-PUFA diet for 1 week. They then measured the participants’ glucose versus fat utilization after an overnight fast. Individuals on a high-PUFA diet burned 23% more glucose and 25% less fat compared to those on a low-PUFA diet. In 2004, Manco et al. (96) wrote that PUFAs lower glucose by increasing GLUT4 transport. In 2013, Gadgil et al. (97) found that increasing PUFA from 8 to 10% of calories for 1 week reduced fasting glucoses.

Although these studies both support the notion that dietary PUFA can increase cellular glucose dependence, this effect was interpreted as a benefit. Thus, both studies have been used incorrectly (in this author’s view) as evidence that PUFA *improves* insulin resistance.

To resolve this apparent discrepancy, it is important to consider the following shortcomings of their perspective. First, it overlooks the broader metabolic consequences of relying on glucose, rather than fat, for energy, especially between meals when blood glucose is limited. Secondly, neither author discussed the possibility that increasing cellular glucose utilization may disrupt whole body glucose homeostasis leading to excessive counter regulatory hormone release. Third, neither author pointed out that their short term studies are incapable of determining longer term impacts. These failings are a predictable outgrowth of the ideology that PUFA are universally beneficial, which has, for many decades, prevented authors from interpreting their results objectively and has prevented others from determining the root cause of metabolic disease.

## Additional evidence linking RBD seed oils to insulin resistance

### Animal studies linking seed oils to insulin resistance

Prolonged elevations of free fatty acids (FFA) have long been observed to impair glucose-stimulated insulin secretion (GSIS), but until 2002, no one had investigated the different effects of differing types of fatty acids. In 2002, Dobbins et al. studied rats fed a diet high in saturated and monounsaturated fats (lard) versus high in PUFA (soy oil). After 4 weeks on the different diets, they tested GSIS and found the insulin sensitivity of “rats consuming Lard diets consistently exceeded that of the soy oil group,” indicating that soy oil in the animals’ diets promoted insulin resistance more powerfully than lard (98).

In 2012, Masi et al. evaluated the effects of two types of high fat diets on C57BL/6 mice, which develop obesity, insulin resistance (IR), diabetes mellitus, advanced fatty liver, and fatty pancreatic diseases when submitted to a high-fat diet. Both groups got a standard high fat diet mainly enriched with saturated fatty acids, but one group was subjected to oral savage with sunflower oil at 2gm/kg twice a week for the duration of the 12 week study. Mice were sacrificed after 12 weeks, and their soleus muscles were subjected to a variety of insulin sensitivity tests. The sunflower oil group had significantly less response to infused insulin, in spite of the fact that the mice subjected to the gavage gained less weight (99).

In 2015, Frances Sladek at the University of California, Riverside, provided important experimental evidence linking dietary soy oil to insulin resistance. Her work in this area began with the observation that soybean oil consumption in the U.S. has dramatically increased over the past century, and that saturated fat intake had not increased significantly, as is commonly stated. She subsequently designed a study that would directly test whether dietary saturated fat or polyunsaturated fat (from RBD soybean oil) promoted more insulin resistance. In a 2015 study, her team demonstrated that mice fed a diet similar in its total PUFA content to that of the average US consumer exhibited greater insulin resistance, weight gain, adiposity, and fatty liver compared to those fed a diet high in saturated fats from coconut oil. This provides direct experimental evidence that the unsaturated fats in soybean oil might be driving insulin resistance (100). Sladek highlighted the role of oxidative stress in driving the liver and mitochondrial damage associated with insulin resistance.

In 2017 Sladek designed a similar study this time with a genetically modified (GM) soybean oil (Plenish^®^), engineered to have lower linoleic acid levels, resembling olive oil’s fatty acid profile. While this GM oil caused less obesity and insulin resistance than conventional soybean oil, it still induced diabetes and fatty liver to a similar degree, indicating that reducing linoleic acid alone does not fully mitigate the oil’s negative effects, suggesting that some of the harms from consuming RBD seed oils emerge from removing nutrients (101).

### Adiposopathy

Adiposopathy, colloquially known as “sick fat” or “inflammatory fat,” is a condition that occurs when adipose cells fail to develop or function normally. Affected fat cells are typically larger than healthy fat cells due to impaired adipogenesis, produce less leptin, fewer adipokines, and “spill” fatty acids into the bloodstream inappropriately (102–104). The link between “sick fat” and excess visceral fat is so strong that some consider the presence of excess visceral fat a surrogate maker for gauging adiposopathy. People with “sick fat” also have more ectopic fat, visceral fat, higher rates of insulin resistance, and greater risk of cardiovascular diseases, cancer, dementia, and complex or fatal infections, including covid-19 (105). Obesity medicine experts have pointed out that adiposopathy is effectively “pathological adipose tissue dysfunction,” suggesting it might warrant reframing as a form of organ failure (106, 107).

A variety of human and animal studies suggest a link between greater RBD seed oil consumption and a variety of hallmarks of adiposopathy. Sladek et al. showed that an RBD oil diet promotes more visceral and ectopic fat formation than coconut oil, which is virtually PUFA-free (100). Human studies detect higher levels of partially oxidized PUFAs in individuals with adiposopathy than in those without (108–110). These compounds also trigger cytokine release, attracting white blood cells to fat tissue and contributing to the hyper coagulable state (111).

## Additional considerations: the elevated glucose “set point”

### The role of the brain in the metabolic tug-of-war

When other cells consume more glucose than usual, the brain may experience relative hypoglycemia, triggering CRH activation even while peripheral blood glucose levels are well are within the normal range. Indeed, people with obesity or type 2 diabetes have otherwise inexplicably lower brain glucose blood levels than peripheral, and this is not the case in lean individuals (112–115). These observations are consistent with a model in which whole body utilization of glucose exceeds the ability of the bloodstream to deliver adequate glucose to the brain under conditions of normoglycemia, and, in response, the brain raises the defended blood glucose level “set point.”

As insulin resistance worsens, blood sugar continues to rise. Initially, blood glucose may only rise slightly, but over time, it increases to the point where physicians diagnose prediabetes. As blood sugar rises, the lows do not dip as sharply, but individuals continue to feel symptoms of low blood sugar during these dips. Eventually, individuals feel hypoglycemic while their glucose levels are technically normal.

### The progression to type 2 diabetes

The final stage involves gradual adjustment of the blood sugar “set point” to progressively higher fasting levels. There is little examination of the concept of blood sugar set point in the literature. This model hypothesizes that downstream consequences of persistent oxidative stress and chronic hepatic gluconeogenesis certainly play important roles. Suffice it to say this last step of the process is complex and poorly understood.

Thus, paradoxically, insulin resistant individuals may feel hypoglycemic even when their blood sugar is high enough to trigger insulin release. This phenomenon is not discussed in the literature, however it is commonly observed among people with type 2 diabetes, as any practicing clinician can attest to. In professional but non-peer review articles, authors invoke a notion of altered set point (116).

Unchecked insulin resistance pushes blood glucose into the range characteristic of full-blown type 2 diabetes. In advanced cases, individuals only feel “normal” when their blood sugar is high, as their baseline glucose rises progressively. As diabetes progresses, both peak and baseline blood sugar levels rise higher and higher. In severe cases, some individuals may go years without achieving normoglycemia even for short time periods.

## Discussion

The Energy First Model has many implications. It directly conflicts with the large majority of literature suggesting saturated fat is the cause of oxidative stress and metabolic diseases. One possible explanation for this apparent shortcoming is that the ideology of saturated fat being a cause of heart disease is actually incorrect. Indeed, several authors have pointed out that the idea was ensconced in government guidelines and taught at universities in spite of the fact that the evidentiary quality is poor (33, 34, 117, 118). Furthermore, evidence from the largest and most well controlled randomized human clinical trial showed that RBD seed oil increased cancer and overall mortality (119). Yet Harvard’s Walter Willet claimed this finding was “an interesting historical footnote that has no relevance to current dietary recommendations” (120). As a result, the unsupported dietary guidelines continue to influence nutrition education and research. If correct, The Energy First Model paints a clear path toward dietary reversal of insulin resistance and other oxidative stress-related diseases.

### The energy model explains a variety of otherwise unexplained phenomena

The framework provided is that insulin resistance stems from oxidative stress that increases the metabolic demand for sugar and gradually shifts the “set point” upward. This framework helps to explain a number of otherwise unexplained or incompletely explained observations. This section will briefly illustrate how several such enigmas fit into the framework.

#### Hypercortisolism

Higher cortisol levels correlate with the severity of insulin resistance as well as its complications (121, 122). This phenomenon is not well explained by current theories of insulin resistance. Component Three of The Energy Model explains how this occurs.

#### The low carb flu

During the first few days to weeks of carbohydrate restriction, many people experience symptoms of fatigue, brain fog, extreme hunger, weakness, and more. These symptoms have been dubbed “the low carb flu” or “keto flu.” Since they often go away, few people have bothered to investigate their origins. It is striking that all these symptoms are also consistent with hypoglycemia and the resultant stress hormone response. When symptoms do go away, it is likely due to a chronic up regulation of gluconeogenesis that may result from the stress hormones thus released. This adaptation would be expected to come at the expense of increased muscle catabolism. Interestingly, Hall et al. noted increased muscle loss during a highly controlled low carb experiment (123).

#### Reduced fat oxidation in type 2 diabetes

Investigators coined the term “metabolic flexibility” to describe the loss of ability to change fuel substrate from carbohydrate to fat. This is a common finding in people with insulin resistance and type two diabetes, as measured by respiratory quotient (124, 125). This finding has been attributed to elevated insulin, which reduces the release of free fatty acids into the circulation (126). But this overlooks the fact people with diabetes and insulin resistance have chronically elevated free fatty acid levels (127). The energy model proposes that increasing glycolysis and reducing fat oxidation are simultaneous adaptive responses the occur frequently in the setting of the modern, oxidative stress inducing diet.

#### The assumption that obese people already have high serum free fatty acids before they develop insulin resistance

People with obesity who do not have type 2 diabetes often have elevated free fatty acid levels (128). This consistent finding has led many authors to assume free fatty acids play a causal role in promoting insulin resistance (127). However this hinges on how insulin resistance is defined. While this article proposes a threshold of greater than 1.0, most authors use values significantly higher than 1.0, which could confuse the understanding of whether insulin resistance develops before or after elevated free fatty acid levels. Furthermore, reduced fat oxidation would be expected to contribute to free fatty acid elevations in the serum.

## Conclusion

The energy model of insulin resistance offers a new framework to understand metabolic disease. Insulin resistance is both nearly ubiquitous in the population and linked to innumerable disease states, thus likely sharing a root cause. The model capitalizes on the large body of evidence identifying oxidative stress as a player in insulin resistance, cancer, and virtually all chronic diseases, and springboards from the assumption that oxidative stress is not incidental but rather a driver of these conditions. By identifying RBD oils as an underappreciated source of excessive dietary PUFA, the model also provides mechanistic rationale that explains the observed near perfect parallel between RBD oil intake and rates of prediabetes and diabetes in the United States. Future researchers should take into account the fact that today’s PUFA intake is out of step with our genetics, and likely to increase our cellular oxidative stress. Because past research has likely suffered from confirmation bias due to the widely accepted notion that RBD oils are “heart healthy,” future researchers should consider setting such notions aside and examining the existing global data at face value.

## Data Availability

The datasets presented in this study can be found in online repositories. The names of the repository/repositories and accession number(s) can be found in the article/Supplementary material.
