# Supplementary material for: The energy model of insulin resistance: A unifying theory linking seed oils to metabolic disease and cancer
Source: Front Nutr. 2025 Apr 29;12:1532961. doi: 10.3389/fnut.2025.1532961 (PMC12105547; doi:10.3389/fnut.2025.1532961)
Supplement: Supplementary file 1 [file Data_Sheet_1.pdf]

Data Sources for Figure 1.

Seed Oil Consumption:

For historical data from 1909 to 1999:

Blasbalg T, Hibbeln JR, Ramsden CE, Majchrzak SF, Rawlings RR. Changes in Consumption of Omega-3 and Omega-6 Fatty Acids in the United States During the 20th Century. Am J Clin Nut. (2011) 93: 950–962.

For consumption of RBD seed oil oils in the United States between 2000 and 2020:

Soybean oil:

Statistica.com Soy oil consumption in the United States from 2000 to 2022 https://www.statista.com/statistics/301037/soybean-oil-consumption-united-states/ [accessed March 15, 2023]

Canola oil:

Statistica.com. Canola oil consumption in the United States from 2000 to 2022 https://www.statista.com/statistics/301036/canola-oil-consumption-united-states/ [accessed March 15, 2023]

Sunflower oil:

Statistica.com Sunflowerseed oil consumption in the United States from 2000 to 2022. https://www.statista.com/statistics/301040/sunflowerseed-oil-consumption-united-states/[accessed March 15, 2023)

Corn oil:

Statistica.com. Domestic consumption of edible corn oil in the United States from 2004/05 to 2023/2024. https://www.statista.com/statistics/1022603/corn-oil-consumption-in-the-us/ [accessed March 15, 2023]

Cottonseed oil:

Statistica.com. Cottonseed oil consumption in the United States from 2000 to 2022. https://www.statista.com/statistics/301029/cottonseed-oil-consumption-united-states/ [accessed March 15, 2023]

Safflower, ricebran, and grapeseed are minor components accounting for less than 5% of total RBD seed oil consumption and data was not readily available for these.

Diabetes Statistics:

CDC.gov National Diabetes Statistics Report (2024) https://www.cdc.gov/diabetes/php/data-research/?CDC_AAref_Val=https://www.cdc.gov/diabetes/pdfs/data/statistics/national-diabetes-statistics-report.pdf[accessed March 27, 2025.

Prediabetes Statistics:

CDC.gov 1 in 3 Americans Have Prediabetes https://www.cdc.gov/diabetes/communication-resources/1-in-3-americans.html

Saturated fat consumption data:

1909-2000

Figure 20 from: Nutrient Content of the U.S. Food Supply, 1909-2000. United States Department of Agriculture, Center for Nutrition Policy and Promotion, Home Economics Research Report Number 56 , November 2004

2000-2020

Shan Z, Rehm CD, Rogers G, Mengan R, Wang DD, Hu FB. Trends in Dietary Carbohydrate, Protein, and Fat Intake and Diet Quality Among US Adults,1999-2016. JAMA. (2019) 322: 1178–1187.
FSRG Dietary Data Briefs [Internet]. Beltsville (MD): United States Department of Agriculture (USDA); 2010-. Dietary Data Brief No. 43Saturated Fat and Food Intakes of Adults. (2022) https://www.ncbi.nlm.nih.gov/books/NBK588575/[accessed March 27, 2025]

Carbohydrate consumption data:

1909-2000
Figure 13 from: Nutrient Content of the U.S. Food Supply, 1909-2000. United States Department of Agriculture, Center for Nutrition Policy and Promotion, Home Economics Research Report Number 56 , November 2004
2000-2020
Our World In Data. Daily caloric supply derived from carbohydrates, protein and fat, United States, 1961 to 2022. (2025) https://ourworldindata.org/grapher/daily-caloric-supply-derived-from-carbohydrates-protein-and-fat[Accessed 3.27.2025]

Red meat consumption data:

Blasbalg TL, Hibbeln JR, Ramsden CE, Majchrzak SF, Rawlings RR. Changes in consumption of omega-3 and omega-6 fatty acids in the United States during the 20th century. Am J Clin Nutr. (2011) 93:950-62.
